# Sustainable Extraction Techniques for Obtaining Antioxidant and Anti-Inflammatory Compounds from the Lamiaceae and Asteraceae Species

**DOI:** 10.3390/foods10092067

**Published:** 2021-09-01

**Authors:** Marisol Villalva, Susana Santoyo, Lilia Salas-Pérez, María de las Nieves Siles-Sánchez, Mónica Rodríguez García-Risco, Tiziana Fornari, Guillermo Reglero, Laura Jaime

**Affiliations:** 1 Institute of Food Science Research (CIAL), Universidad Autónoma de Madrid (CEI UAM+CSIC), 28049 Madrid, Spain; marisol.villalva@uam.es (M.V.); susana.santoyo@uam.es (S.S.); maria.siles@uam.es (M.d.l.N.S.-S.); monica.rodriguez@uam.es (M.R.G.-R.); tiziana.fornari@uam.es (T.F.); guillermo.reglero@uam.es (G.R.); 2 Faculty of Accounting and Administration, Universidad Autónoma de Coahuila, Fco. Javier Mina 150, Luis Echeverría Álvarez Sector Norte, 27085 Torreón, Coahuila, Mexico; lsalas@uadec.edu.mx; 3 Imdea-Food Institute, Universidad Autónoma de Madrid (CEI UAM+CSIC), 28049 Madrid, Spain

**Keywords:** *Achillea millefolium*, *Origanum majorana*, anti-inflammatory activity, antioxidant activity, sustainable extraction, phenolic composition

## Abstract

*Melissa officinalis* L. and *Origanum majorana* L., within Lamiaceae family, and *Calendula officinalis* L. and *Achillea millefolium* L., within the Asteraceae, have been considered a good source of bioactive ingredients with health benefits. In this study, the supercritical fluid extraction (SFE) using pure CO_2_, and the ultrasound assisted extraction (UAE) were proposed as green techniques to obtain plant-based extracts with potential antioxidant and anti-inflammatory activities. Higher values of total phenolic content and antioxidant activity were achieved in UAE ethanol:water (50:50, *v/v*) extracts. Meanwhile, UAE pure ethanol extracts showed greater anti-inflammatory activity. RP-HPLC-PAD-ESI-QTOF-MS/MS analysis showed a vast number of phenolic compounds in the extracts, including unreported ones. *O. majorana* ethanol:water extract presented the highest content of phenolics and antioxidant activity; among its composition, both rosmarinic acid and luteolin glucoside derivatives were abundant. The pure ethanol extract of *A. millefolium* resulted in an important content of caffeoylquinic acid derivatives, luteolin-7-*O*-glucoside and flavonoid aglycones, which could be related to the remarkable inhibition of TNF-α, IL-1*β* and IL-6 cytokines. Besides, borneol and camphor, found in the volatile fraction of *A. millefolium*, could contributed to this latter activity. Thus, this study points out that *O. majorana* and *A. millefolium* are considered a promising source of bioactive ingredients with potential use in health promotion.

## 1. Introduction

In the last decades, an increasing number of studies have focused on finding new sources of food ingredients for applications in pharmaceuticals, cosmetics, the food industry and, of course, nutrition [1]. Several compounds, such as phenolic compounds, terpenes, carotenoids, saponins and peptides are considered to possess interesting biological activities [2]. The first step in the development of food ingredients involves finding out the most suitable plant matrix to draw those bioactivities. In this regard, the Lamiaceae and Asteraceae families represent a great source of valued plant species. These species are often used as flavoring ingredients in many culinary preparations, but are also considered as medicinal herbs, where their beneficial effects have been mainly related to phenolic compounds [3,4].

Phenolic compounds, which are the secondary metabolites of plants, have been extensively tested as antibacterial, antitumor, antioxidant and anti-inflammatory agents [5]. Because of their structural characteristics, phenolic compounds are considered as natural antioxidant and anti-inflammatory chemicals which are able to avoid cellular damage related to oxidative stress. Moreover, phenolic compounds are considered to be capable of reducing the occurrence of many chronic pathologies and diseases, such as cardiovascular and neurodegenerative disease, and cancer [6].

*Melissa officinalis* L., commonly known as lemon balm, is a species of Lamiaceae typically used for the prevention and treatment of nervous disturbances and gastrointestinal disorders. Different extracts of *M. officinalis* have exhibited antioxidant, antimicrobial and anti-inflammatory activities [7,8]. In particular, hydroxycinnamic acids, such as rosmarinic acid and luteolin glycosylated derivatives, were found as the main phenolic constituents [9]. *Origanum majorana* L., also known as marjoram, is another species of Lamiaceae that has been appreciated because of its therapeutic properties against gastrointestinal, respiratory and neurological disorders. Extracts from marjoram, including its essential oil, have been reported to possess antimicrobial, antioxidant, anti-proliferative and anti-inflammatory activities [4,6]. Caffeic acid derivatives and flavonoids, along with ursolic acid, carvacrol and terpineol, have been associated with these biological activities [1,6].

The Asteraceae species *Achillea millefolium* L., usually known as yarrow, is consumed worldwide for the treatment of gastrointestinal disorders and hepatobiliary complaints, as well as for wound/ulcer healing and skin inflammation [10]. Essential oil from *A. millefolium* has been associated with antimicrobial and anti-inflammatory activities [3,11]. Moreover, alcoholic and water extracts have shown antioxidant and antitumor properties [12,13]. Caffeic acid derivatives, mainly caffeoylquinic acids and flavones, have been reported as parts of the yarrow’s composition [10]. *Calendula officinalis* L., (marigold) has been frequently used for healing skin diseases, wounds and duodenal ulcers [14,15]. Extracts of this Asteraceae species has been reported for their anti-inflammatory, antibacterial, antioxidant and antitumor activity [16,17]. Quercetin derivatives, mainly their glycosylated and methylated forms, have been described within the phenolic composition of marigold [18].

Nowadays, according to the sustainable development goals launched by the United Nations, the use of sustainable extraction techniques to obtain plant-based bioactive ingredients is essential [19]. For that purpose, extraction techniques run by the *Green Chemistry* principles should be used. Supercritical Fluid Extraction (SFE) has been widely used for the extraction of hydrophobic compounds in a more efficient and green environment [1]. SFE primarily uses CO_2_ as carrier, as well as a green and GRAS (Generally Recognized as Safe) solvent, which allows the recovery of high-quality extracts. Besides, SFE represents a minimizing solvent-consuming process due to CO_2_ recirculation. Ultrasound Assisted Extraction (UAE) applies ultrasonic vibrations generated at high frequencies to enhance the extraction of plant bioactives. Compared to conventional techniques, UAE has been considered as more efficient due to its solvent-reduction consumption and shorter-extraction times [20]. Therefore, minor solvent and energy waste are achieved. Methanol and/or its water mixtures have also been frequently used. Nevertheless, this toxic solvent should be replaced with other GRAS solvents, such as ethanol, to obtain high-quality food ingredients.

Therefore, this study focused on the use of advanced and sustainable extraction methodologies (SFE-CO_2_ and UAE using ethanol and water) to optimize bioactive secondary metabolite extraction from *Melissa officinalis* L. (MEL), *Origanum majorana* L. (MAJ), *Achillea millefolium* L. (MIL) and *Calendula officinalis* L. (CAL). Moreover, a screening of the antioxidant and anti-inflammatory activities, along with a comprehensive HPLC-PAD-ESI-QTOF-MS/MS and GC-MS analysis of the extracts, was carried out to correlate the composition and bioactivities.

## 2. Materials and Methods

### 2.1. Reagents

HPLC grade acetonitrile was obtained from Macron Fine Chemicals (Madrid, Spain) and formic acid (99%) from Acros Organics (Madrid, Spain). Pure ethanol (99.5%) was purchased from Panreac (Barcelona, Spain). 2,2′-Azobis (2-methylpropionamidine) dihydrochloride (AAPH), 2,2′-Azino-bis (3-ethylbenzothiazoline-6-sulfonic acid) diammonium salt (ABTS), fluorescein, 3-(4,5-dimethylthiazol-2-yl)-2,5-diphenyl tetrazolium bromide (MTT), phorbol 12-myristate 13-acetate (PMA), and (±)-6-Hydroxy-2,5,7,8-tetramethylchromane-2-carboxylic acid (Trolox, 97%) were purchased from Sigma-Aldrich (Madrid, Spain). Phenolic acids authentic standards (HPLC purity ≥ 95%) were purchased from Sigma-Aldrich, Phytolab (Madrid, Spain), Cymit Química SL (Madrid, Spain) and Extrasynthese S.A. (Genay, France) as detailed in Supplementary Material (Table S1).

### 2.2. Plant Material and Extraction

*Origanum majorana* L. (leaves), *Melissa officinalis* L. (leaves), *Achillea millefolium* L. (inflorescences and leaves), and *Calendula officinalis* L. (flowers) were purchased as dry raw material from a local herbal company (Murciana Herboristería, Murcia, Spain). The samples were ground at 9000 rpm for 60 s to diminish the particle size (Grindomix GM200, Retsch GmbH, Haan, Germany), preserved under vacuum and stored at room temperature until their use.

#### 2.2.1. Ultrasound Assisted Extraction (UAE)

UAE plant extracts were obtained using a Branson 250 digital device (Branson Ultrasonics, Danbury, CT, USA) with an electric power of 200 W and frequency of 60 Hz. A corresponding solvent volume of ethanol or ethanol:water (50:50, *v/v*) was added to 35 g of ground plant material in a ratio of 1:10 (plant/solvent, *w/v*). The mixture was submitted to ultrasounds for 30 min and 70% amplitude using a probe of ½′ diameter. Next, samples were filtered, and ethanol was removed under vacuum at 30 °C (IKA RV 10, Madrid, Spain). Samples were freeze-dried when required. Dried extracts were maintained at −20 °C protected from light until use. Extractions were carried out in triplicate.

#### 2.2.2. Supercritical Fluid Extraction (SFE)

SFE assays were carried out in a pilot-plant supercritical fluid extractor (Model SF2000, Thar Technology, Pittsburgh, PA, USA) equipped with a 2 L cylinder extraction cell. This cell was fully filled with plant material, i.e., 713 g for *M. officinalis* L., 550 g for *O. majorana* L., 500 g for *C. officinalis* L. and 383 g for *A. millefolium* L. To obtain the SFE extracts, a CO_2_ flow was established at 70 g/min at 140 bar and 40 °C. After the extraction process (180 min), a precipitated oleoresin-type extract was recovered from the extraction vessel with ethanol. Then, ethanol was removed under vacuum (30 °C) to obtain a final solid residue, which was kept at −20 °C until use. Extractions were conducted in triplicate.

### 2.3. Total Phenolic Content and Antioxidant Activity

The total phenolic content (TPC) was determined by the Folin-Ciocalteu colorimetric method, as described by Singleton et al. [21]. A calibration curve of gallic acid was used, and results are expressed as mg of gallic acid equivalents (GAE) per gram of extract.

To determine the antioxidant activity of the extracts, two methodologies were used. The ABTS^•+^ radical scavenging capacity was performed at four different concentrations of each extract, following the procedure described by Re et al. [22]. Results are expressed in mmol of Trolox equivalents/g of extract. The Oxygen Radical Absorbance Capacity (ORAC) assay was carried out according to Huang y col. [23]. The reaction occurred in a 96-well black round-bottom plate containing 150 µL of fluorescein stock solution (8 × 10^−8^, PBS 0.075 M), 25 µL of plant extract, PBS (blank samples) or Trolox solution (reference standard), and 25 µL of AAPH radical fresh solution (165.94 mM). Excitation and emission wavelength were set at 485 nm and 520 nm, respectively (Infinite M200, Tecan, Madrid, Spain), and the fluorescence intensity was recorded every 1 min at 37 °C until the value was <5% of the initial reading. Results are expressed as mmol of Trolox/g of extract. Analyses were done in triplicate.

### 2.4. Cell Culture and Anti-Inflammatory Activity

Human THP-1 monocytes (ATCC, Manassas, VA, USA) were maintained and cultured in supplemented RPMI 1640 media (Gibco, Paisley, UK) until confluence to perform the cytotoxicity and anti-inflammatory assays as described by Villalva et al. [24]. For differentiation of THP-1 monocytes to macrophages, cells were seeded in 24-well plates (5 × 10^5^ cells/mL) with 100 ng/mL of PMA for 48 h. The cytotoxicity of the extracts was tested (10, 20 and 50 using µg/mL) on differentiated macrophages using 3-(4,5-dimethylthiazol-2-yl)-2,5-diphenyl tetrazolium Bromide (MTT). For anti-inflammatory assays, macrophages were incubated with or in absence of 0.05 µg/mL of bacterial lipopolysaccharide (LPS from *E. coli* O55:B5, Sigma-Aldrich, Madrid, Spain) in the presence of a non-cytotoxic extract concentration for 24 h. The secretion of the pro-inflammatory cytokines, TNF-α, IL-1*β* and IL-6, was measured in the cell’s supernatants using ELISA assay kits (BD Biosciences, Aalst, Belgium), according to the manufacturer’s instructions. Results were expressed as mean of three determinations ± standard deviation.

### 2.5. RP-HPLC-PAD-ESI-QTOF-MS/MS Analysis

Mass spectrometric detection was performed using an HPLC system (1100 series, Agilent Technologies, Santa Clara, CA, USA) connected to an ultra-high-resolution QTOF instrument (MAXIS II, Bruker, Bremen, Germany). Electrospray ionization source was used in the negative mode for all the analyses, and the parameters were adjusted as follows: capillary voltage 3400 V, end plate offset 500 V, in-source Collision Induced Dissociation energy (isCID) 30 eV for MS/MS spectra. Nitrogen was used as the nebulizer gas (pressure of 4 Bar) and drying gas (heated to 250 °C, flow 8 L/min). For accurate high-resolution, mass spectrometry (HRMS) internal calibration was performed after each chromatographic run using a mixture of phosphazenes. The accurate obtained masses were processed using the elemental composition calculator incorporated in the data analysis software (Bruker, Bremen, Germany). Prior to mass detection, dry extracts were dissolved at 2.5 mg/mL in ethanol:water (60:40, *v/v*) and filtered using a 0.45 μm polyvinylidene fluoride (PVDF) filter. Separation was achieved in a reversed-phase ACE Excell 3 Super C18 column (150 mm × 4.6 mm, 3 μm, ACT, Aberdeen, Scotland) protected by a pre-column (ACE 3 C18-AR, 10 mm × 3 mm), as described by Villalva et al. [24], with slight modifications. Briefly, the chromatographic separation was performed using solvent A (water with 0.1% formic acid, *v/v*) and solvent B (pure acetonitrile), following the next gradient elution: 0–1 min, 5% B; 6 min, 15% B; 21 min, 25% B; 26 min, 35% B; 36–41 min, 50% B; 43–48 min, 100% B; 50 min, 5% B. Identification of phenolic compounds was performed by contrasting the accurate mass and MS/MS fragmentation pattern with the literature, and by comparison of its retention time and UV-Vis max absorption pattern with the available analytical standards. Phenolic compounds quantification was carried out in a HPLC-PAD system (1260 Infinity series, Agilent Technologies, Santa Clara, CA, USA) using a five-level calibration curve of authentic phenolics standards. Those compounds for which standards were not available were quantified with the calibration curve of that compound with the greater structural affinity, e.g., apigenin-7-*O*-glucoside was used for apigenin glycosylated derivative, and chlorogenic acid was used for caffeoylquinic acid derivative. In addition, ethyl gallate was used as internal standard. Analyses were carried out by triplicate, and the results are expressed as mg phenolic compound/g of extract.

### 2.6. GC-MS Identification

The volatile fraction of the extracts was determined by GC-MS in an Agilent Technologies 7890A system (Agilent Technologies, Santa Clara, CA, USA) coupled to a 5975C triple-axis mass spectrometer detector. The column used was a HP-5MS (5% Phenyl methyl siloxane, Agilent 19091S-433) and the chromatographic conditions for separation were followed as described by García-Risco et al. [25]. The components were identified based on their relative retention time and mass spectrum compared to the library data of the GC-MS system (NIST MS 2.0). Analyses were carried out in triplicate.

### 2.7. Statistical Analysis

Statistical analysis was performed by one-way analysis of variance (ANOVA) followed by Fisher’s least significance differences (LSD) test to discriminate among means (*p* < 0.05). To determine the correlation between the different experimental data, a Pearson test was carried out (*p* < 0.05). Statgraphics Centurion XVI (Statpoint Technologies Inc., Warrenton, VA, USA) software was used for this purpose.

## 3. Results

### 3.1. Evaluation of TPC and Antioxidant Activity of the Plant Extracts

Two different green extraction methodologies involving SFE and UAE were used in this study to obtain the bioactive compounds from the Lamiaceae and Asteraceae species, following the extraction conditions previously studied by our research group [25]. As shown in Table 1, the extraction yield was influenced by the solvent polarity regardless of the plant used. Overall, the SFE extracts demonstrated a lower mass recovery in comparison with the UAE. Moreover, within the UAE extracts, those obtained with pure ethanol achieved significantly inferior yield values than the extracts obtained with 50% ethanol.

Two complementary assays, ABTS radical scavenging and ORAC, were used to evaluate the antioxidant activity of the SFE and UAE extracts (Table 1). Overall, the solvent polarity seemed to exert a clear influence on the antioxidant activity despite the plant matrix. Thus, an increment in the solvent polarity allowed us to obtain extracts with higher antioxidant activity. In this context, the greatest TEAC and ORAC values were achieved using 50% ethanol (UAE-50), followed by 100% ethanol (UAE-100) extracts. In contrast, SFE extracts, carried out with pure CO_2_, presented a limited antioxidant activity. Moreover, both the UAE-50 extracts from Lamiaceae species, MAJ and MEL, presented a higher antioxidant activity than those from the Asteraceae species, highlighting *O. majorana* as the most effective and *C. officinalis* as the least effective.

A similar behavior was found regarding the TPC, as can be observed in Table 1. For all the plants studied, the extracts obtained by UAE presented higher TPC values than those obtained by SFE. Moreover, the UAE-50 extracts exhibited a significantly higher recovery of phenolic compounds (mg GAE/g extract) compared to 100% ethanol, as reported by Rodríguez-Pérez et al. [20] regarding the UAE plant extracts. Therefore, we confirmed the close correlation between the total content of TPC and the antioxidant activity (TEAC or ORAC value) (Supplementary Material, Figure S1). Moreover, despite of the extraction technique and the solvent used, the greatest TPC value corresponded to MAJ, while the lowest was found in CAL.

Overall, within the studied extracts, the *Lamiacea* species exhibited a greater performance regarding the TPC and antioxidant activity properties. The radical scavenging capacities of extracts from *O. majorana* and *M. officinalis* have already been related to their high phenolic content [9,26].

### 3.2. Anti-Inflammatory Activity of the Plant Extracts

**Figure 1:**
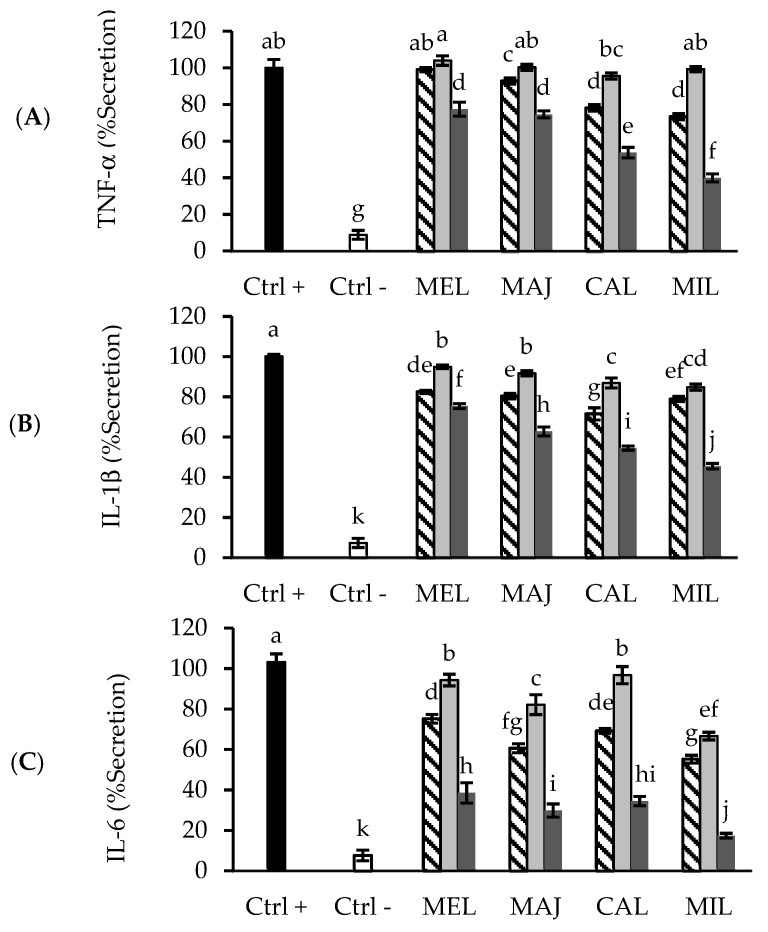
Levels of (**A**) TNF-α, (**B**) IL-1*β* and (**C**) IL-6 secreted by THP-1 macrophages, activated with LPS, in presence of 10 μg/mL of SFE (stripped bars), UAE-50 (light grey bars) and UAE-100 (dark grey bars) plant extracts. Ctrl+, positive control (cells stimulated with LPS without extract). Ctrl −, negative control (cells non-stimulated). MEL, *Melissa officinalis* L., MAJ, *Origanum majorana* L., CAL, *Calendula officinalis* L, MIL, *Achillea millefolium* L. Each bar is the mean of three determinations ± SD. ^a–k^ Different letters indicate statistical differences among LSD procedure (*p* < 0.05).

To evaluate the anti-inflammatory activity of the Lamiaceae and Asteraceae species, LPS-activated THP-1 macrophages were exposed to 10 μg/mL of SFE and UAE extracts, which represented a non-cytotoxic concentration (data not shown). A significant release of the studied proinflammatory cytokines was observed in the stimulated THP-1 macrophages (positive control) in contrast to the non-stimulated cells (negative control) (Figure 1).

The addition of plant extracts from both techniques, SFE or UAE, resulted in dissimilar results, regarding the release inhibition of the three cytokines studied. Thus, the most relevant inhibition of TNF-α was observed in presence of the UAE-100 extracts, for all plant species (Figure 1A). Moreover, the highest inhibition was exhibited by MIL UAE-100 followed by CAL UAE-100, demonstrating a reduction approx. 60% and 50%, respectively, compared with the positive control. However, none of the UAE-50 extracts inhibited this cytokine secretion. Concerning SFE extracts, only CAL and MIL presented a secretion reduction of approx. 30%.

The obtained results for IL-1*β* release in presence of plant extracts (Figure 1B) were similar to those obtained for TNF-α, since UAE-100 extracts were the most active. In particular, MIL UAE-100 produced an important decrease in the IL-1*β* secretion (approx. 55%) compared with the positive control. Finally, UAE-100 extracts also exhibited an important inhibition of IL-6 (Figure 1C), especially MIL UAE-100 demonstrated approx. an 80% inhibition. In addition, for this cytokine, all SFE extracts presented a moderate inhibition. Overall, these results bring to light the higher anti-inflammatory potential of the studied Asteraceae species compared to the Lamiaceae ones, mainly UAE-100 extracts.

According to the above results, a trend for proinflammatory cytokines inhibition could be delineated, i.e., UAE-100 > SFE > UAE 50 extracts. Thus, contrary to the antioxidant activity, the anti-inflammatory activity of these extracts was not completely related to their TPC. Therefore, the observed anti-inflammatory properties of the extracts could be linked to the presence of specific phenolic compounds [14]. However, the contributions of other types of compounds cannot be discarded, since the anti-inflammatory effect of terpenes, presented in the essential oil of these plants, has been described [4,11].

### 3.3. RP-HPLC-PAD-ESI-QTOF-MS/MS Identification and RP-HPLC-PAD Quantification of Selected UAE Extracts

As mentioned previously, a close relationship between TPC and antioxidant activity has been established. Nevertheless, to better understand which specific compounds are behind this activity, a comprehensive analysis of such extracts with a remarkable activity, i.e., UAE-50 extracts, was carried out. Although several studies regarding the phenolic composition of the four studied plants have already published, in this study, 146 compounds were tentatively identified, of which 26 compounds have not been previously described for these plant matrices. Figure S2 shows the base peak chromatogram of the *A. millefolium* UAE-50 extract, where each peak is numbered according to its elution order. Furthermore, Table 2 shows the overall results of the retention time (Rt), theoretical mass (*m/z*), accurate mass (*m/z*) and MS/MS fragmentation patterns of all identified compounds, i.e., organic acids, phenolic acids (hydroxybenzoic and hydroxycinnamic acids and their derivatives), flavonoids (flavones, flavonols, flavanones and their derivatives), other polar compounds and saponins.

Although some similarities were found among all UAE-50 extracts, such as the presence of organic acids and caffeic acid, we observed many differences. Within the Lamiaceae species, 49 compounds were identified in *M. officinalis*, mostly rosmarinic acid (RA) and its derivatives, either dimers, trimers or tetramers. In total, 70 compounds were identified in *O. majorana* showing a multivariate composition of caffeic acid derivatives and flavonoids, mainly glycosylated flavones. Besides, flavanones were only detected in this latter plant. With respect to the Asteraceae species, 41 compounds were identified in *C. officinalis*. Glycosylated and methylated derivatives of flavonols, along with caffeic acid derivatives, were particularly exhibited in this extract. Finally, within the 59 compounds identified in *A. millefolium*, the presence of several mono- and di- caffeoylquinic acid (CQA) derivatives represented a special characteristic in this plant, along with a great variety of methoxylated and glycosylated flavones.

Different studies have already published the compositions, mainly the phenolic composition, of *M. officinalis* [7,8,9] and *O. majorana* [5,26,27]. However, an extended characterization of the ethanol:water extracts, including the unreported compounds, was undertaken the present article for these matrices (Table 2). Regarding the phenolic acid constituents, some yunnaneic acid isomers have been described for *M. officinalis* [7,8]. Nevertheless, to the best of our knowledge, yunnaneic acid D (peak 64) is still unreported. Similarly, sagecoumarins have been identified in Lamiaceae species, mainly in *S. officinalis* and *M. officinalis* [8,28], but not in *O. majorana*. Thus, peak 118 was tentatively identified as a sagecoumarin isomer in MAJ-50 according to its fragmentation pattern [8].

This study also contributes to the understanding of an extended flavonoid composition of *O. majorana*. To the best of our knowledge, 11 compounds were not yet referenced in marjoram, despite the fact that they were found in other *Lamiaceae* spp. For instance, the presence of luteolin diglucuronide (peak 44), eriodictyol hexoside (peak 69) and isorhamnetin hexoside (peak 85) were described in *T. vulgaris* [29]; eriocitrin (peak 59) and kaempferide glucururonide (peak 99) were reported in *Origanum* spp. [30,31]; and 6-hydroxyluteolin-7-*O*-glucoside (peak 53) and luteolin acetylglucoside (peak 93) were detected in *S. officinalis* [28,32]. Furthermore, in the MAJ-50 extract, a group of methoxylated flavonoids was detected and tentatively identified as dihydroxyquercetin dimethyl ether (peak 133), trihydroxy dimethoxyflavone (peak 138), methoxyacacetin (peak 144) and dihydroxy trimethoxyflavone (peak 145). This type of methoxylated compounds has been also characterized in thyme using LC–MS/MS techniques [33].

Regarding the Asteraceae species, the composition of *C. officinalis* agrees with the published literature [15,17,18]. However, no previous records were found for kaempferol-3-*O*-rutinoside (peak 80), which was also identified by the comparison with its authentic standard. Moreover, according to our results, a considerable number of phenolic compounds remained unreported in *C. officinalis* (peaks 10, 14, 16, 19, 34, 35, 49, 50 and 68). Nevertheless, it is worth mentioning that these phenolic compounds have recently been recognized in other *Calendula spp* and *Asteracea* spp. [13,16,34]. In CAL-50, the presence of saponins derivatives is a noteworthy characteristic, since calendasaponins B, A and G have recently been reported in other *Calendula* spp. [16], where peaks 108, 122 and 135 were tentatively identified, respectively. The composition of *A. millefolium*, has been extensively addressed due to a wide range of bioactivities found in this plant. Thus, most of the compounds derived from our study were in accordance with those found previously [10,12,13]. In addition, new CQA derivatives were defined in the *A. millefolium* ethanol:water extract, namely feruloyl-*O*-caffeoylquinic acid (peak 111) and tricaffeoylquinic acid (peak 119). In this sample, the presence of a new flavone derivate was also observed, corresponding with 6-hydroxyluteolin-7-*O*-glucoside (peak 53). This derivate has not been reported specifically in *A. millefolium*, but it was characterized within the Asteraceae genera [32].

From all tentatively identified compounds, 107 phenolic compounds were quantified in the ethanol:water extracts, as can be observed in Table 3. Within the Lamiaceae species, rosmarinic acid (RA) was particularly abundant in MEL-50, while for MAJ-50 extract, RA and 6-hydroxyluteolin-7-*O*-glucoside were found as the main phenolics. With respect to *Asteraceae*, isorhamnetin-3-*O*-rhamnosylrutinoside was the most representative constituent for CAL-50, whereas 3,5-dicaffeoylquinic acid (3,5-DCQA) was the most representative for MIL-50.

**Figure 2:**
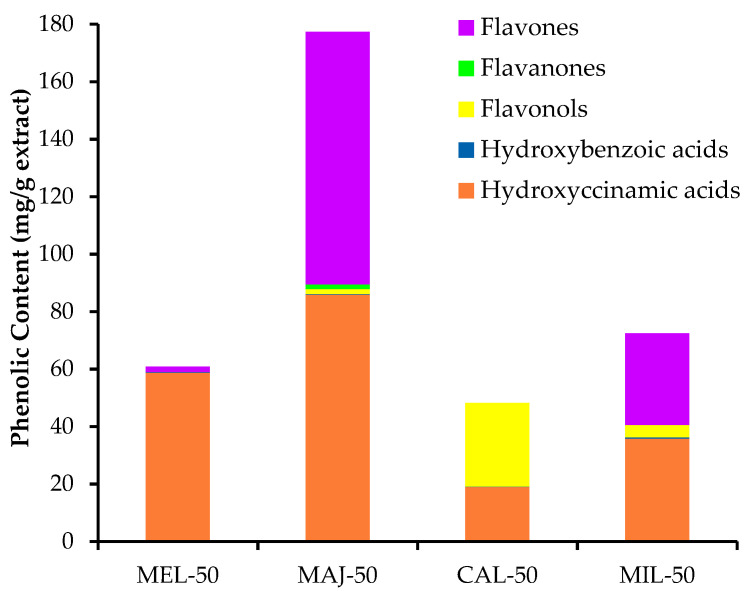
Concentration by type of phenolic compounds in the studied UAE-50 extracts, expressed as mg/g extract. MEL, *Melissa officinalis* L., MAJ, *Origanum majorana* L., CAL, *Calendula officinalis* L., MIL, *Achillea millefolium* L.

Nevertheless, in an attempt to better understanding the quantitative differences within the studied samples, the cumulative concentration of phenolic compounds in the four extracts was plot, as shown in Figure 2. Considering these results, the antioxidant activity of the ethanol:water extracts could be attributable to the sum of the phenolic compounds (mg/g extract), but also to the presence of specific phenolic compounds. Thus, the greatest antioxidant activity of MAJ-50 was clearly associated to its maximum content of phenolic compounds (Figure 2), mainly hydroxycinnamic acids and flavones, compared to the other extracts, but probably influenced by those most representative compounds in this sample, such as RA and its dimers, i.e., lithosphermic acid isomer, and luteolin glucosylated derivatives, i.e. 6-hydroxyluteolin-7-*O*-glucoside, luteolin-*O*-glucoside and luteolin-7-*O-β*-glucoside, which also have been recognized for its antioxidant activity [9,32].

Although MEL-50 showed a smaller total accumulation of phenolics than MIL-50 (Figure 2), *M. officinalis* showed better results than *A. millefolium* in terms of antioxidant activity (Table 1). In this regard, the main difference was the greater concentration of hydroxycinnamic acids in MEL-50. Therefore, its better antioxidant effect could be related. Thus, the presence of a high content of RA and its derivatives in MEL-50 could be considered the principal factor contributing to its antioxidant activity.

Regarding MIL-50 and CAL-50, although both plants belong to the same family, their individual phenolic composition showed quite different results (Figure 2). MIL-50 was distinguished by the presence of a similar content of both hydroxycinnamic acids and flavones, whereas in CAL-50, the abundance of flavonols was relevant. A greatest content of 3,5-DCQA, along with chlorogenic acid, was found in *A. millefolium* ethanol:water extract. This may be primarily attributed to its antioxidant activity [35]. Although this type of antioxidant component, i.e., CGA and DCQAs, was also found in CAL-50, its lack of antioxidant activity could be attributed to its low content of phenolic compounds overall.

In addition, the total phenolic composition of the MIL-100 extract (Table 3), which exhibited the highest anti-inflammatory activity, displayed a quite superior content compared to the *A. millefolium* ethanol:water extract. In this context, significant increases were found in certain compounds, such as 3,5-DCQA, luteolin-7-*O*-glucoside, casticin and centaureidin. In agreement with our results, Ali et al. [10] reported the anti-inflammatory activity of an enriched fraction in DCQAs and flavonoids from *A. millefolium.* At the same time, centaureidin and casticin have been associated with anti-inflammatory activity [36,37]. Furthermore, other aglycones that were enriched in the MIL-100 extract, such as apigenin, quercetin, luteolin and diosmetin, seemed to reduce the IL-6 and TNF-α secretion in the LPS-stimulated RAW macrophages [38]. Thus, the cytokines release inhibition shown by the MIL-100 extract could be attributed, at least in part, to the abovementioned phenolic compounds. On the other hand, since pure ethanol was used as the solvent extract, volatile compounds typically found in *A. millefolium* essential oil with powerful anti-inflammatory properties may have also been extracted [3,11]. Therefore, a GC-MS analysis of the MIL-100 extract was also conducted.

### 3.4. GC-MS Composition of Ethanolic Extracts

The composition of the volatile fraction of MIL UAE-100 extract, shown in Table 4, demonstrated the presence of a wide range of monoterpenes and sesquiterpenes [3], found in a greater or lesser extent. As can be observed, borneol (23.8%) was the most abundant compound so far, although camphor (10.4%), and *β*-eudesmol (8.6%) were also representative in the extract. As mentioned by other authors, borneol and camphor, some of the mainly constituents in several *Asteracea* spp. essential oil, significantly inhibited nitric oxide production, CCL2 (Chemokine (C-C motif) ligand 2) release and cytokines (TNF-α, IL-1*β* and IL-6) secretion in macrophages [4,11].

With respect to the the anti-inflammatory properties of *A. millefolium* ethanol extract, an exclusive influence of one group of compounds cannot be completely established. However, some accumulative factors, such as (*i*) a greater abundance of some specific phenolics, (*ii*) the presence of terpenoid compounds in the volatile fraction and (*iii*) the accumulative, or synergistic, effect of minor compounds, could contribute to this activity.

## 4. Conclusions

This study highlights that the application of innovative and sustainable techniques for obtaining a specific group of bioactive compounds, together with the use of advance chemical analysis of complex plant extracts, makes it possible to obtain new healthy ingredients. Thereby, UAE with ethanol:water or pure ethanol proved to be a better sustainable methodology than SFE with pure CO_2_ for obtaining compounds with antioxidant and/or anti-inflammatory activities from the four studied plants. Moreover, *Origanum majorana* (Lamiaceae) was displayed as the best source of antioxidant compounds, whereas *Achillea millefolium* (Asteraceae) showed a remarkable anti-inflammatory activity on LPS-stimulated macrophages. A deep high-resolution MS/MS analysis of the plant extracts constituents, particularly phenolic compounds, allowed the tentative identification of the compounds responsible of these bioactivities. Thus, the antioxidant activity of *O. majorana* extracts may be manly linked to its high content of phenolic compounds, especially rosmarinic acid and luteolin glucoside derivates. On the other hand, the anti-inflammatory activity of *A. millefolium* could, in part, be explained by its richness of specific phenolics, i.e., 3,5-DCQA, luteolin-7-*O*-glucoside, casticin and centaureidin, but also to the presence of volatile compounds, such as borneol and camphor. Thus, both, *O. majorana* and *A. millefolium* should be considered as good sources of potent bioactive ingredients with promising applications in multiple industry, especially the food and pharmaceutical industries.

## Figures and Tables

**Table 1:** Extraction yield, total phenolic content (TPC) and antioxidant activity of the plant extracts, obtained by supercritical fluids extraction (SFE) and ultrasound assisted extraction (UAE) techniques.

		MEL	MAJ	CAL	MIL
**Yield ^1^**	SFE	0.6 ± 0.1 ^C^_d_	1.5 ± 0.2 ^C^_b_	2.8 ± 0.2 ^C^_a_	0.8 ± 0.1 ^C^_c_
	UAE-100	4.2 ± 0.2 ^B^_c_	5.9 ± 0.4 ^B^_a_	6.7 ± 0.7 ^B^_a_	5.1 ± 0.1 ^B^_b_
	UAE-50	22.7 ± 0.6 ^A^_b_	15.6 ± 0.1 ^A^_c_	27.9 ± 0.6 ^A^_a_	14.8 ± 0.1 ^A^_d_
**TPC ^2^**	SFE	16 ± 1 ^C^_b_	56 ± 1 ^C^_a_	13 ± 1 ^C^_c_	14 ± 2 ^B^_bc_
	UAE-100	70 ± 1 ^B^_c_	148 ± 3 ^B^_a_	36 ± 1 ^B^_d_	111 ± 2 ^A^_b_
	UAE-50	112 ± 2 ^A^_b_	247 ± 5 ^A^_a_	82 ± 2 ^A^_d_	106 ± 3 ^A^_c_
**TEAC ^3^**	SFE	0.03 ± 0.01 ^C^_c_	0.64 ± 0.02 ^C^_a_	0.05 ± 0.02 ^B^_c_	0.08 ± 0.01 ^C^_b_
	UAE-100	0.24 ± 0.01 ^B^_c_	0.71 ± 0.01 ^B^_a_	0.06 ± 0.01 ^B^_d_	0.29 ± 0.03 ^B^_b_
	UAE-50	0.71 ± 0.02 ^A^_b_	1.46 ± 0.03 ^A^_a_	0.33 ± 0.02 ^A^_d_	0.52 ± 0.04 ^A^_c_
**ORAC ^4^**	SFE	0.76 ± 0.01 ^C^_b_	1.59 ± 0.04 ^C^_a_	0.38 ± 0.02 ^C^_c_	0.73 ± 0.02 ^C^_b_
	UAE-100	0.94 ± 0.03 ^B^_c_	2.57 ± 0.14 ^B^_a_	0.48 ± 0.01 ^B^_d_	1.86 ± 0.11 ^B^_b_
	UAE-50	2.71 ± 0.12 ^A^_b_	5.17 ± 0.09 ^A^_a_	1.32 ± 0.10 ^A^_d_	2.16 ± 0.02 ^A^_c_

^1^ Yield expressed in percentage (%). ^2^ TPC as mg GAE/g extract. ^3^ TEAC (Trolox Equivalent Antioxidant Capacity) as mmol Trolox/g extract. ^4^ ORAC (Oxygen Radical Absorbance Capacity) as mmol Trolox/g extract. ^A–D^ Different superscript letters denote significant differences within a column (*p* < 0.05). _a–c_ Different subscript letters denote significant differences within a line (*p* < 0.05). MEL, *Melissa officinalis*. MAJ, *Origanum majorana*. MIL, *Achillea millefolium*. CAL, *Calendula officinalis*.

**Table 2:** Compounds identified by HPLC-PAD-ESI-QTOF-MS/MS (negative ionization mode) in the selected UAE 50% ethanol extracts from the Lamiaceae and Asteraceae genera.

Peak No.	Rt (min)	Compound	Theoretical Mass *(m/z)*	Accurate Mass *(m/z)*	MS/MS Product Ions (*m/z*)	Extract
***Organic acids***						
1	2.5	Gluconic acid	195.0510	195.0511	129 (100)	M, O, C, A
2	3.0	Quinic acid	191.0561	191.0564	127 (100), 111 (30)	M, O, C, A
3	3.3	Tartaric acid	149.0092	149.0093	72 (100)	M, O
4	3.5	Malic acid	133.0142	133.0144	115 (100)	M, O, C, A
5	3.7	Citric acid	191.0197	191.0192	111 (100), 87 (40)	O, C, A
6	4.3	Isocitric acid	191.0197	191.0192	111 (100), 87 (40)	O
7	5.1	Succinic acid	117.0930	117.0950	73 (100)	M
***Hydroxybenzoic acids and derivatives***						
9	5.3	Gallic acid *	169.0142	169.0144	125 (100)	M, O
10	5.4	Protocatechuic acid pentoside	285.0616	285.0622	153 (100), 109 (80)	C
11	5.5	3,4-dihydroxyphenillactic acid—hexoside	359.0984	359.0987	197 (100), 179 (60)	M, O
13	5.7	Dihydroxybenzoic acid hexoside	315.0722	315.0721	153 (98), 109 (80)	M, O, A
15	6.1	3,4-dihydroxyphenil-lactic acid	197.0455	197.0456	179 (80), 135 (100)	M, O
17	6.4	Hydroxybenzoic acid hexoside	299.0772	299.0774	137 (100)	M, O, C
20	8.2	Protocatechuic acid *	153.0193	153.0197	109 (100)	M, O, A
49	19.4	Syringic acid derivative	313.0565	313.0572	197 (100), 121 (25)	O, C
***Hydroxyccinamic acids and derivatives***						
12	5.6	Vainillic acid hexoside	329.0772	329.0767	167(100), 152 (20)	O
14	6.0	Hydroxyferulic acid hexoside isomer I	371.0620	371.0627	209 (100), 191 (55)	C
16	6.2	Hydroxyferulic acid hexoside isomer II	371.0620	371.0627	209 (100), 191 (40)	C
18	6.8	Neochlorogenic acid *	353.0878	353.0877	191 (100), 179 (76), 135 (40)	O, C, A
19	7.1	Hydroxyferulic acid hexoside isomer III	371.0620	371.0627	209 (100), 191 (25)	C
21	8.5	Caftaric acid isomer	311.0409	311.0410	179 (14), 149 (100)	A
22	8.6	Caffeic acid dihexoside	503.1406	503.1410	179 (14), 149 (100)	M
23	8.8	Caftaric acid *	311.0409	311.0410	179 (14), 149 (100)	M, A
25	9.5	Caffeoylquinic acid isomer I	353.0878	353.0877	191 (100), 179 (20)	A
26	9.6	Caffeic acid hexoside I	341.0878	341.0882	179 (100) 135 (60)	M, C
27	9.8	Coumaric acid hexoside	325.0929	325.0934	163 (100), 119 (20)	M, O
28	10.2	Chlorogenic acid *	353.0878	353.0877	191 (100), 161 (10)	C, A
29	10.3	Cryptochlorogenic acid *	353.0878	353.0877	191 (100), 161 (11)	O, C, A
30	10.8	Caffeic acid hexuronide	355.0671	355.0678	191 (100)	C
32	13.9	Caffeic acid hexoside II	341.0878	341.0882	179 (100), 135 (65)	M, O
33	14.1	Coumaric acid pentoside	295.0459	295.0464	163 (80), 119 (63)	M
34	14.3	Coumaroylquinic acid	337.0929	337.0936	191 (100)	C
35	14.5	Caffeoylshikimic acid	335.0772	335.0780	179 (100), 135 (77)	C
36	14.8	Caffeoylquinic acid isomer II	353.0878	353.0879	191 (100), 161 (10)	C, A
37	15.2	Feruloyl tartaric acid	325.0565	325.0571	193, 161, 134	M
39	15.8	Caffeic acid *	179.0350	179.0353	135 (100)	M, O, C, A
46	18.8	Yunaneic acid E isomer I	571.1093	571.1099	329 (31), 197 (100), 179 (24), 135 (39)	M
48	19.2	Yunaneic acid E isomer II	571.1093	571.1099	329 (31), 197 (100), 179 (24), 135 (39)	M
50	19.7	Feruloylquinic acid	367.1035	367.1042	191 (100), 173 (40)	C
54	20.5	Salvianolic acid I	537.1038	537.1043	493 (25), 339(100), 295(22), 197(60)	M, O
64	21.9	Yunnaneic acid D isomer	539.1195	539.1198	197 (80), 135 (60)	M
76	24.9	Rosmarinic acid hexoside	521.1301	521.1305	359 (21), 197 (100), 179 (12), 161 (29)	M, O
81	26.5	Chicoric acid	473.0725	473.0730	311 (50), 179 (80), 149 (100), 135 (10)	M
82	26.9	3,4-dicaffeoylquinic acid *	515.1195	515.1189	353 (100), 335 (30), 179 (69), 173 (80)	A
86	28.0	1,5-dicaffeoylquinic acid *	515.1195	515.1190	353 (100), 191 (40)	C, A
88	28.1	Sagerinic acid	719.1618	719.1619	539 (20), 521 (7), 359 (100), 197 (20)	M
89	28.3	3,5-dicaffeoylquinic acid *	515.1195	515.1190	353 (100), 191 (55), 179 (35), 135 (21)	C, A
90	28.4	Salvianolic acid E isomer	717.1456	717.1463	537 (28), 519 (100), 339 (88), 295 (22)	M, O
95	28.9	Isorosmarinic acid	359.0772	359.0771	197 (35), 179 (30), 161 (100), 135 (20)	M
98	29.4	4,5-dicaffeoylquinic acid *	515.1195	515.1190	353 (100), 191 (10), 179 (30), 173 (40)	C, A
101	30.1	Rosmarinic acid *	359.0772	359.0771	197 (80), 179 (50), 161 (100), 135 (30)	M, O
104	30.5	Lithospermic acid *	537.1038	537.1041	493 (44), 359 (43), 295 (93), 161 (100)	O
***Hydroxyccinamic acids and derivatives (continued)***						
105	30.7	Lithospermic acid isomer I	537.1038	537.1041	493 (24), 295 (80), 197 (18), 161 (90)	M, O
106	30.8	Salvianolic acid B *	717.1461	717.1445	519 (30), 359 (100), 295 (10), 179 (10)	M, O
109	31.5	Dicaffeoylquinic acid isomer	515.1195	515.1191	353 (100), 191 (15),179 (35), 173 (30)	C, A
110	31.7	Lithospermic acid isomer II	537.1038	537.1040	493 (32), 359 (30), 295 (100)	M, O
111	31.9	Feruloyl-*O*-caffeoylquinic acid	529.1351	529.1350	367 (100), 193 (55), 191 (22)	A
112	32.2	Salvianolic acid L isomer I	717.1461	717.1464	519 (100), 359 (30), 339 (15), 149 (18)	M, O
113	32.4	Sagecoumarin caftaride	829.1258	829.1260	667 (88), 535 (80), 311 (50), 135 (47)	M
114	32.5	Salvianolic acid L hydroxycaffeide	895.1730	895.1728	519 (77), 369 (73), 161 (100)	M
115	32.6	Salvianolic acid A isomer	493.1140	493.1145	359 (100), 295 (10), 197 (20), 161 (39)	M, O
116	32.9	Methylrosmarinic acid	373.0929	373.0932	179 (100), 161 (20), 135 (82)	M
118	33.4	Sagecoumarin isomer	535.0882	535.0886	359 (8), 197 (10), 177 (100), 161 (19)	M, O
119	33.7	Tricaffeoylquinic acid	677.1512	677.1499	515 (100), 353 (68)	C, A
120	33.7	Salvianolic acid C derivative	715.1305	715.1304	535 (100), 491 (11), 311 (9), 135 (7)	M
123	34.4	Salvianolic acid L isomer II	717.1461	717.1462	519 (100), 339 (13)	M, O
128	36.3	Rosmarinic acid derivative I	565.1351	565.1353	359 (60), 197 (28), 179 (20), 161 (100)	M, O
130	37.9	Rosmarinic acid derivative II	565.1351	565.1353	359 (100), 197 (30), 179 (13), 135 (36)	M
136	39.7	Salvianolic acid C isomer	491.0984	491.0986	267 (16), 179 (100), 161 (21)	M, O
139	40.0	Salvianolic acid F isomer	313.0718	313.0720	161 (100)	M
140	40.2	Rosmarinic acid derivative III	565.1351	565.1353	359 (94), 197 (24), 179 (20), 161 (100)	M, O
143	42.7	Salvianolic acid C caffeoylhydroxycaffeide	849.1672	849.1674	359 (100), 179 (6), 161 (20), 135 (40)	M
***Flavone derivatives***						
24	9.3	Luteolin 6,8-*C*-dihexoside	609.1461	609.1453	489 (100), 325 (40)	O, A
31	13.7	Vicenin 2 *	593.1512	593.1507	473 (100)	O, A
38	15.5	Apigenin-hexoside-pentoside I	563.1406	563.1401	473 (10), 443 (20)	A
40	17.9	Schaftoside isomer	563.1406	563.1401	473 (10), 443 (20)	A
41	18.1	Schaftoside *	563.1406	563.1401	473 (10), 443 (20)	O, A
42	18.2	Luteolin-*C*-hexoside	447.0933	447.0925	357 (38), 327 (100)	O
44	18.4	Luteolin diglucuronide	637.1046	637.1042	285(100)	O, A
45	18.8	Homoorientin *	447.0933	447.0930	429 (30), 357 (100), 327 (80)	A
47	19.2	Luteolin-hexoside-hexuronide	623.1254	623.1246	447 (90), 285 (100), 112 (60)	A
51	19.7	Apigenin-hexoside-pentoside II	563.1406	563.1401	473 (10), 443 (20)	A
52	19.8	Luteolin dihexoside I	609.1461	609.1451	447 (100), 357 (26), 327 (70), 285 (10)	A
53	20.2	6-Hydroxyluteolin-7-*O*-glucoside	463.0882	463.0880	301 (100)	O, A
56	20.7	Luteolin hexoside	447.0871	447.0873	285 (100)	O
55	20.8	Apigenin dihexoside	593.1512	593.1511	269 (100)	A
62	21.8	Luteolin dihexoside II	609.1745	609.1750	447 (20), 285(10)	O, A
63	21.9	Luteolin rutinoside	593.1432	593.1430	285 (100)	O
70	24.0	Apigenin deoxylhexoside	577.1563	577.1557	269 (100)	A
72	24.4	Luteolin-*O*-glucoside	447.0931	447.0932	285 (100), 151 (20)	O
73	24.5	Apigenin glycosylated derivative	445.1140	445.1136	269 (100)	A
74	24.8	Luteolin-7-*O-β*-glucoside *	447.0933	447.0928	285 (100)	M, O, A
77	25.2	Luteolin-7-*O*-glucuronide *	461.0725	461.0722	285 (100)	O, A
78	25.4	Apigenin hexoside	431.0928	431.0928	269 (100)	O
91	28.6	Diosmin *	607.1663	607.1668	607 (10), 299 (100), 284 (10)	O
93	28.7	Luteolin acetylglucoside	489.0970	489.0973	447 (30), 285 (100)	O
94	28.8	Apigenin-7-*O*-glucoside *	431.0984	431.0980	269 (100)	O, A
96	29.0	Luteolin-*O*-malonylglucoside	533.0937	533.0931	489 (100), 285 (15)	A
97	29.4	Apigenin-7-*O*-glucuronide *	445.0776	445.0768	269 (100)	O
100	29.8	Apigenin-*O*-hexuronide	445.0776	445.0775	269 (100)	A
102	30.3	Luteolin-*O*-hexuronide	461.0725	461.0727	285 (100)	M
121	34.1	Luteolin dimer	569.0725	569.0718	285 (100), 112 (80)	A
122	34.8	Luteolin *	285.0405	285.0400	175 (80), 151 (100), 107 (51)	M, O, A
132	38.1	Apigenin *	269.0455	269.0454	112 (100)	O, A
137	39.7	Diosmetin *	299.0561	299.0554	112 (100)	A
138	39.8	Trihydroxy dimethoxyflavone I	329.0667	329.0665	314 (20), 299 (100)	O
141	40.6	Trihydroxy dimethoxyflavone II	329.0667	329.0665	299 (100)	A
144	43.1	Methoxyacacetin	313.0718	313.0716	283 (100), 112 (60)	O, A
145	43.4	Dihydroxy trimethoxyflavone	343.0823	343.0820	328 (100), 313 (20)	O, A
***Flavonol derivatives***						
43	18.2	Quercetin-3-*O*-rhmanosylrutinoside	755.2040	755.2052	301 (100), 271 (23)	C
57	20.8	Quercetin-3-neohesperidoside	609.1461	609.1468	301 (100)	C
58	21.0	Isorhamnetin-3-*O*-rhamnosylrutinoside*	769.2197	769.224	315 (100), 300 (20)	C
60	21.4	Quercetin hexoside I	463.0882	463.0880	301 (100)	A
61	21.7	Quercetin-*O*-pentosylhexoside	595.1305	595.1310	301 (100)	C
65	22.9	Rutin *	609.1097	609.1093	301 (100)	C, A
66	23.1	Isovitexin	431.0984	431.0981	311 (100)	A
67	23.2	Vitexin *	431.0984	431.0981	311 (100)	A
68	23.4	Quercetin-malonylhexosyl-rhamnoside	695.1465	695.1472	651 (100), 301 (23)	C
71	24.1	Isorhamnetin-3-*O*-neohesperoside	623.1618	623.1621	315 (100), 300 (10)	C
75	24.9	Quercetin hexoside II	463.0882	463.0889	301 (100)	C
79	25.5	Quercetin hexuronide	477.0675	477.0671	301 (100)	A
80	25.9	Kaempferol-3-*O*-rutinoside *	593.1512	593.1521	285 (100)	C
83	27.4	Ishoramnetin-3-*O*-rutinoside *	623.1618	623.1627	315 (100)	C
84	27.7	Quercetin-3-*O*-acetyl-glucoside	505.0988	505.0996	463 (30), 301 (100)	C
85	27.8	Isorhamnetin hexoside I	477.1038	477.1035	315 (100)	O, A
87	28.0	Quercetin pentoside	433.072	433.0718	301 (100)	O
92	28.7	Isorhamnetin-3-*O*-glucoside *	477.1038	477.1045	315 (100)	C
99	29.8	Kaempferide glucuronide	475.0819	475.0821	299.0522 (100)	O
103	30.3	Ishoramnetin-3-*O*-acetylglucoside	519.1144	519.1150	315 (100), 300(15)	C
107	30.9	Isorhamnetin hexoside II	477.1038	477.1035	315 (100)	A
125	35.0	Quercetin *	301.0354	301.0352	151 (60)	O, A
126	36.1	Methoxyquercetin isomer	315.0510	315.0508	301 (100)	O, A
127	36.2	Quercetin dimethyl ether	329.0624	329.0625	314 (100), 299 (70)	O
129	37.5	Jaceidin isomer	359.0767	359.0727	344 (57), 329 (100)	O
133	38.3	Dihydroxyquercetin dimethyl ether	331.0823	331.0817	299 (100)	O
134	38.3	Isorhamnetin *	315.0510	315.0511	301 (100), 209 (15)	C
142	41.0	Centaureidin	359.0772	359.0770	344 (59), 229 (100)	A
146	45.8	Casticin *	373.0929	373.0923	358 (43), 343 (90)	A
***Flavanone derivatives***						
59	21.2	Eriocitrin	595.1585	595.1591	287(100)	O
69	23.5	Eriodyctiol hexoside	449.1029	449.1028	287.0524 (100)	O
117	33.3	Eriodictyol	287.0561	287.0555	151 (100), 135 (85)	O
131	37.9	Naringenin *	271.0612	271.0607	151 (100)	O
***Other compounds***						
8	5.2	Arbutin *	271.0823	271.0819	108 (100)	O
108	31.3	Calendasaponin B	971.4857	971.4855	971 (100), 809 (40)	C
122	34.2	Calendasaponin A	1117.5436	1117.5439	1117 (100), 955 (10)	C
135	39.5	Calenduloside G	793.4373	793.4376	631 (100), 613 (30)	C

* Comparison with standard. Rt, retention time. M, Melissa officinalis; O, Origanum majorana; C, Calendula officinalis; A, Achillea millefolium.

**Table 3:** Phenolic composition of the selected UAE extracts. MEL, *Melissa officinalis* L., MAJ, *Origanum majorana* L., CAL, *Calendula officinalis* L., MIL, *Achillea millefolium* L. Results are expressed in mean ± S.D. (mg/g extract).

Compound	MEL-50	MAJ-50	CAL-50	MIL-50	MIL-100
***Hydroxybenzoic acids and derivatives***					
Protocatechuic acid pentoside	nd	nd	0.14 ± 0.03	nd	nd
3,4-dihydroxyphenil lactic acid	0.61 ± 0.04	1.29 ± 0.06	nd	nd	nd
Protocatechuic acid *	0.19 ± 0.07	0.22 ± 0.10	nd	0.35 ± 0.09 ^1^	0.11 ± 0.03
Syringic acid derivative	nd	<LOQ	0.38 ± 0.04	nd	nd
***Hydroxyccinamic acids and derivatives***					
Hydroxyferulic acid hexoside isomer I	nd	nd	0.16 ± 0.05	nd	nd
Hydroxyferulic acid hexoside isomer II	nd	nd	0.20 ± 0.08	nd	nd
Neochlorogenic acid *	nd	0.27 ± 0.05	0.60 ± 0.10	0.43 ± 0.10 ^1^	0.26 ± 0.05
Hydroxyferulic acid hexoside isomer III	nd	nd	0.24 ± 0.01	nd	nd
Caftaric acid isomer	nd	nd	nd	0.08 ± 0.03	0.06 ± 0.03
Caffeic acid dihexoside	0.32 ± 0.05	nd	nd	nd	nd
Caftaric acid *	0.38 ± 0.08	nd	nd	0.16 ± 0.03 ^1^	0.08 ± 0.03
Caffeoylquinic acid isomer I	nd	nd	nd	0.22 ± 0.04	0.19 ± 0.04
Caffeic acid hexoside I	<LOQ	nd	0.20 ± 0.01	nd	nd
Chlorogenic acid *	nd	nd	7.92 ± 0.39	7.84 ± 0.57 ^1^	6.41 ± 0.33
Cryptochlorogenic acid *	nd	0.75 ± 0.11	0.16 ± 0.04	0.47 ± 0.09^1^	0.13 ± 0.06
Caffeic acid hexoside II	0.21 ± 0.08	0.29 ± 0.07	nd	nd	nd
***Hydroxyccinamic acids and derivatives (continued)***					
Coumaroylquinic acid	nd	nd	0.09 ± 0.03	nd	nd
Caffeoylshikimic acid	nd	nd	0.20 ± 0.05	nd	nd
Caffeoylquinic acid isomer II	nd	nd	<LOQ	0.64 ± 0.10 ^1^	0.10 ± 0.06
Caffeic acid *	0.57 ± 0.09	0.78 ± 0.10	0.19 ± 0.04	0.40 ± 0.07	0.33 ± 0.04
Yunnaneic acid E isomer I	1.20 ± 0.15	nd	nd	nd	nd
Yunnaneic acid E isomer II	1.94 ± 0.14	nd	nd	nd	nd
Salvianolic acid I	0.77 ± 0.13	<LOQ	nd	nd	nd
Yunnaneic acid D isomer	0.37 ± 0.05	nd	nd	nd	nd
Rosmarinic acid hexoside	7.19 ± 0.84	<LOQ	nd	nd	nd
Chicoric acid	0.75 ± 0.06	nd	nd	nd	nd
3,4-dicaffeoylquinic acid *	nd	nd	nd	1.42 ± 0.09	1.43 ± 0.07
1,5-dicaffeoylquinic acid *	nd	nd	0.31 ± 0.09	2.57 ± 0.10 ^1^	1.73 ± 0.09
Sagerinic acid	2.22 ± 0.18	nd	nd	nd	nd
3,5-dicaffeoylquinic acid *	nd	nd	5.37 ± 0.96	15.30 ± 1.02	21.93 ± 1.21 ^1^
Salvianolic acid E	0.43 ± 0.05	<LOQ	nd	nd	nd
Isorosmarinic acid	0.46 ± 0.05	nd	nd	nd	nd
4,5-dicaffeoylquinic acid *	nd	nd	2.61 ± 0.15	5.70 ± 0.20 ^1^	4.41 ± 0.13
Rosmarinic acid *	19.21 ± 1.02	37.61 ± 1.90	nd	nd	nd
Lithospermic acid *	nd	10.52 ± 0.71	nd	nd	nd
Lithospermic acid isomer I	1.84 ± 0.09	21.30 ± 1.02	nd	nd	nd
Salvianolic acid B *	0.49 ± 0.02	2.06 ± 0.08	nd	nd	nd
Dicaffeoylquinic acid isomer	nd	nd	0.08 ± 0.03	0.18 ± 0.06 ^1^	0.08 ± 0.02
Lithospermic acid isomer II	8.65 ± 0.52	3.56 ± 0.23	nd	nd	nd
Feruloyl-*O*-caffeoylquinic acid	nd	nd	nd	0.13 ± 0.03	0.12 ± 0.02
Salvianolic acid L isomer I	1.75 ± 0.08	4.32 ± 0.15	nd	nd	nd
Sagecoumarin caftaride	0.64 ± 0.06	nd	nd	nd	nd
Sagecoumarin isomer	0.40 ± 0.05	1.33 ± 0.09	nd	nd	nd
Tricaffeoylquinic acid	nd	nd	0.13 ± 0.02	0.30 ± 0.06	0.39 ± 0.07
Salvianolic acid C derivative	2.19 ± 0.09	nd	nd	nd	nd
Salvianolic acid L isomer II	0.95 ± 0.06	0.98 ± 0.07	nd	nd	nd
Rosmarinic acid derivative I	<LOQ	0.27 ± 0.07	nd	nd	nd
Rosmarinic acid derivative II	0.30 ± 0.03	nd	nd	nd	nd
Salvianolic acid F isomer	1.16 ± 0.09	nd	nd	nd	nd
Rosmarinic acid derivative III	0.46 ± 0.03	0.35 ± 0.04	nd	nd	nd
Salvianolic acid C caffeoylhydroxycaffeide	3.32 ± 0.10	nd	nd	nd	nd
***Flavone derivatives***					
Vicenin 2 *	nd	2.56 ± 0.17	0.11 ± 0.06	4.00 ± 0.33 ^1^	2.35 ± 0.13
Apigenin-hexoside-pentoside I	nd	nd	nd	0.55 ± 0.21 ^1^	0.44 ± 0.12
Schaftoside isomer	nd	nd	nd	3.20 ± 0.14 ^1^	1.38 ± 0.10
Schaftoside *	nd	<LOQ	nd	2.73 ± 0.12 ^1^	2.04 ± 0.12
Luteolin-*C*-hexoside	nd	2.19 ± 0.15	nd	nd	nd
Homoorientin *	nd	nd	nd	1.00 ± 0.17	2.26 ± 0.22 ^1^
Apigenin-hexoside-pentoside II	nd	nd	nd	2.02 ± 0.18 ^1^	1.22 ± 0.12
Luteolin dihexoside I	nd	nd	nd	1.82 ± 0.10	3.01 ± 0.12 ^1^
6-hydroxyluteolin-7-*O*-glucoside	nd	35.80 ± 2.11	nd	1.99 ± 0.09	2.48 ± 0.10
Apigenin dihexoside	nd	nd	nd	0.31 ± 0.07	0.23 ± 0.06
Luteolin dihexoside II	nd	<LOQ	nd	0.27 ± 0.04	0.26 ± 0.06
Apigenin deoxylhexoside	nd	nd	nd	0.26 ± 0.09	0.36 ± 0.04
Luteolin-*O*-glucoside	nd	17.52 ± 1.10	nd	nd	nd
Apigenin glycosylated derivative	nd	nd	nd	3.21 ± 0.13 ^1^	2.42 ± 0.09
Luteolin-7-*O*-*β*-glucoside *	0.21 ± 0.03	14.61 ± 1.01	nd	4.96 ± 0.35	8.23 ± 0.72 ^1^
***Flavone derivatives (continued)***					
Luteolin-7-*O*-glucuronide *	nd	4.09 ± 0.11	nd	0.69 ± 0.07	0.82 ± 0.07
Diosmin *	nd	3.77 ± 0.19	nd	nd	nd
Apigenin-7-*O*-glucoside *	nd	2.17 ± 0.09	nd	1.05 ± 0.21	2.28 ± 0.32 ^1^
Luteolin-O-malonylglucoside	nd	nd	nd	0.26 ± 0.07	0.55 ± 0.09 ^1^
Apigenin-7-*O*-glucuronide *	nd	1.70 ± 0.09	nd	nd	nd
Luteolin-*O*-hexuronide	1.64 ± 0.11	nd	nd	nd	nd
Luteolin *	<LOQ	1.10 ± 0.08	nd	1.70 ± 0.07	1.89 ± 0.09 ^1^
Apigenin *	nd	0.09 ± 0.02	nd	0.42 ± 0.04	0.59 ± 0.05 ^1^
Diosmetin *	nd	nd	nd	0.34 ± 0.03	0.45 ± 0.05 ^1^
Trihydroxy dimethoxyflavone I	nd	0.69 ± 0.07	nd	nd	nd
Trihydroxy dimethoxyflavone II	nd	nd	nd	<LOQ	0.24 ± 0.06
Methoxyacacetin	nd	0.04 ± 0.02	nd	<LOQ	0.22 ± 0.05
Dihydroxy trimethoxyflavone	nd	nd	nd	0.13 ± 0.03	0.31 ± 0.04 ^1^
***Flavonol derivatives***					
Quercetin-3-*O*-rhmanosylrutinoside	nd	nd	1.17 ± 0.73	nd	nd
Quercetin 3-neohesperidoside	nd	nd	0.18 ± 0.06	nd	nd
Isorhamnetin-3-*O*-rhamnosylrutinoside *	nd	nd	14.22 ± 1.30	nd	nd
Quercetin hexoside I	nd	nd	nd	0.72 ± 0.09	1.34 ± 0.90 ^1^
Quercetin-*O*-pentosylhexoside	nd	nd	0.36 ± 0.07	nd	nd
Rutin *	nd	nd	0.57 ± 0.06	0.99 ± 0.09	1.12 ± 0.11
Isovitexin	nd	nd	nd	0.50 ± 0.09	0.46 ± 0.07
Vitexin *	nd	nd	nd	0.46 ± 0.09	0.66 ± 0.11
Quercetin-malonylhexosyl-rhamnoside	nd	nd	0.65 ± 0.12	nd	nd
Isorhamnetin-3-*O*-neohesperidoside	nd	nd	1.89 ± 0.14	nd	nd
Quercetin hexoside II	nd	nd	0.19 ± 0.06	nd	nd
Quercetin hexuronide	nd	nd	nd	0.45 ± 0.04 ^1^	0.21 ± 0.03
Kaempferol-3-*O*-rutinoside*	nd	nd	0.19 ± 0.07	nd	nd
Ishoramnetin-3-*O*-rutinoside *	nd	nd	7.23 ± 0.62	nd	nd
Quercetin-3-*O*-acetyl-glucoside	nd	nd	0.64 ± 0.04	nd	nd
Isorhamnetin hexoside I	nd	<LOQ	nd	0.48 ± 0.07	1.18 ± 0.10 ^1^
Isorhamnetin-3-*O*-glucoside*	nd	nd	0.67 ± 0.09	nd	nd
Ishoramnetin-3-*O*-acetylglucoside	nd	nd	1.12 ± 0.09	nd	nd
Isorhamnetin hexoside II	nd	nd	nd	0.17 ± 0.04	0.53 ± 0.07 ^1^
Quercetin *	nd	0.36 ± 0.03	nd	0.33 ± 0.04	0.64 ± 0.05 ^1^
Methoxyquercetin isomer	nd	<LOQ	nd	0.58 ± 0.08	0.82 ± 0.11 ^1^
Quercetin dimethyl ether	nd	0.81 ± 0.10	nd	nd	nd
Jaceidin isomer	nd	0.62 ± 0.08	nd	nd	nd
Dihydroxyquercetin dimethyl ether	nd	0.61 ± 0.07	nd	nd	nd
Centaureidin	nd	nd	nd	0.27 ± 0.06	1.90 ± 0.10 ^1^
Casticin *	nd	nd	nd	0.35 ± 0.03	2.53 ± 0.11 ^1^
***Flavanone derivatives***					
Eriodictyol	nd	0.67 ± 0.05	nd	nd	nd
Naringenin *	nd	0.94 ± 0.10	nd	nd	nd
**Σ Phenolic compounds**	60.8 ± 1.30	176.2 ± 2.5	48.2 ± 0.9	72.4 ± 1.0	83.2 ± 1.0 ^1^

* Identified and quantified via comparison with authentic standards. nd, not detected. < LOQ, below limit of quantification. ^1^ Indicates statistical differences between MIL-50 and MIL-100 extracts (*p* < 0.05).

**Table 4:** Chemical composition of the peak area contribution of *Achillea millefolium* L. ethanolic extract (UAE-100 extract) of the volatile fraction identified by GC-MS.

Rt (min)	RI	Compound	% Area
4.6	997	Yomogi alcohol	6.1
5.1	1028	Eucalyptol	5.0
5.5	1058	Artemisia ketone	4.3
5.8	1079	Artemisia alcohol	5.6
6.2	1101	Thujone	2.9
6.9	1138	Camphor	10.4
7.2	1160	Borneol	23.8
7.6	1174	Terpinene-4-ol	1.9
8.7	1261	(*5E*)-5,9-Dimethyl-5,8-decadien-2-one	2.9
9.3	1299	Carvacrol	2.9
12.1	1478	α-curcumene	0.9
13.5	1569	Spathulenol	2.7
13.6	1578	Caryophillene oxide	2.6
13.8	1589	Viridiflorol	5.1
14.3	1630	δ-Cadinol	4.6
14.5	1640	*β*-Eudesmol	8.6
15.6	1718	Chamazulene	5.9
16.8	1890	Corymbolone	3.8
		**∑ AUC**	**4.10 × 10^6^**

Rt: retention time. RI: retention index. AUC: area under curve.

## Supplementary Materials

The following are available online at https://www.mdpi.com/article/10.3390/foods10092067/s1, Table S1: Authentic commercial standards (HPLC purity ≥ 95%), Figure S1: Antioxidant activity of Asteraceae and Lamiaceae plant extracts as a function of TPC (mg GAE/g). (**A**) TEAC value (mmol trolox/g); (**B**) ORAC (mmol trolox/g). Figure S2: Base-peak chromatogram of *Achillea millefolium* obtained by UAE (ethanol-water, 50:50, *v/v*) analysed by RP-HPLC-ESI-QTOF-MS in negative ionization mode. * IS, internal standard (ethyl gallate).
