# Supplementary material for: Sustainable Extraction Techniques for Obtaining Antioxidant and Anti-Inflammatory Compounds from the Lamiaceae and Asteraceae Species
Source: Foods. 2021 Sep 1;10(9):2067. doi: 10.3390/foods10092067 (PMC8472344; doi:10.3390/foods10092067)
Supplement: Supplementary file 1 [file foods-10-02067-s001.zip › foods-1326499-supplementary.pdf]

Sustainable extraction techniques for obtaining antioxidant and anti-inflammatory compounds from Lamiaceae and Asteraceae genera

M. Villalva[1,*], S. Santoyo[1], L. Salas-Pérez[2], M. N. Siles-Sánchez[1], M. R. García-Risco[1], T. Fornari[1], G. Reglero[1,3], L. Jaime[1,*]

[1] Institute of Food Science Research (CIAL). Universidad Autónoma de Madrid (CEI UAM+CSIC). 28049 Madrid, Spain.

[2] Polytechnic University of Gomez Palacio (UPGOP). 35120 Durango, México.

[3] Imdea-Food Institute, Universidad Autónoma de Madrid (CEI UAM+CSIC), 28049 Madrid, Spain.

*Corresponding authors:

M.V. Institute of Food Science Research (CIAL). Universidad Autónoma de Madrid. Nicolás Cabrera, 9. 28049 Madrid, Spain. E-mail: marisol.villalva@uam.es Telephone: +34 910 017 900

L.J. Institute of Food Science Research (CIAL). Universidad Autónoma de Madrid. Nicolás Cabrera, 9. 28049 Madrid, Spain. E-mail: laura.jaime@uam.es Telephone: +34 910 017 925

SUPPLEMENTARY MATERIAL

Table S1. Authentic commercial standards (HPLC purity ≥95%)

Supplier	Authentic standard
Cymit Química SL, Madrid, Spain	Ishoramnetin-3-*O*-rutinoside
	Isorhamnetin-3-*O*-glucoside
	Isorhamnetin-3-*O*-rhamnosylrutinoside
Extrasynthese S.A., Genay, France	Apigenin-7-*O*-glucuronide
	Apigenin-7-*O*-glucoside
	Caffeic acid
	Ethyl gallate
	Homoorientin
	Isoquercitrin
	Isorhamnetin
	Luteolin-7-*O*-*β*-glucoside
	Luteolin-7-*O*-glucuronide
	Quercetin
	Rutin
Phytolab, Madrid, Spain	1,5- Dicaffeoylquinic acid
	3,4- Dicaffeoylquinic acid
	3,5- Dicaffeoylquinic acid
	4,5- Dicaffeoylquinic acid
	Apigenin
	Caftaric acid
	Casticin
	Cryptochlorogenic acid
	Diosmin
	Kaempferol-3-*O*-rutinoside
	Lithospermic acid
	Luteolin
	Naringenin
	Neochlorogenic acid
	Orientin
	Protocatechuic acid
	Salvianolic acid B
	Schaftoside
	Vicenin II
Sigma-Aldrich, Madrid, Spain	Arbutin
	Chlorogenic acid
	Diosmetin
	Gallic acid
	Rosmarinic acid
	Vitexin

(A)

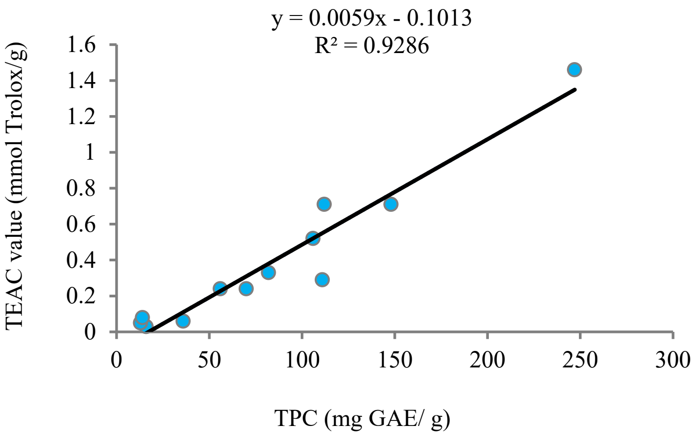

(B)

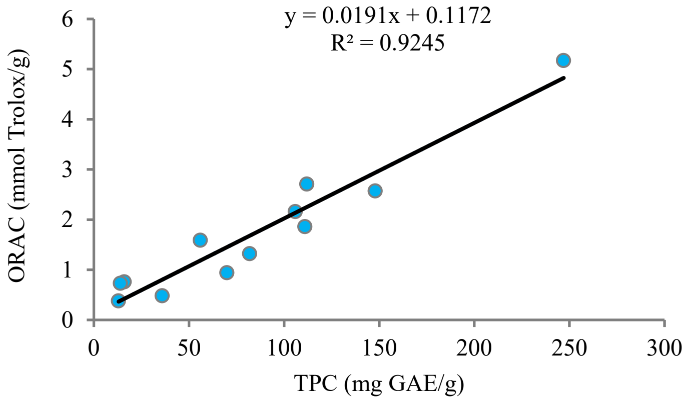

Figure S1. Antioxidant activity of Asteraceae and Lamiaceae plant extracts as a function of TPC (mg GAE/ g). (A) TEAC value (mmol trolox/ g); (B) ORAC (mmol trolox / g).

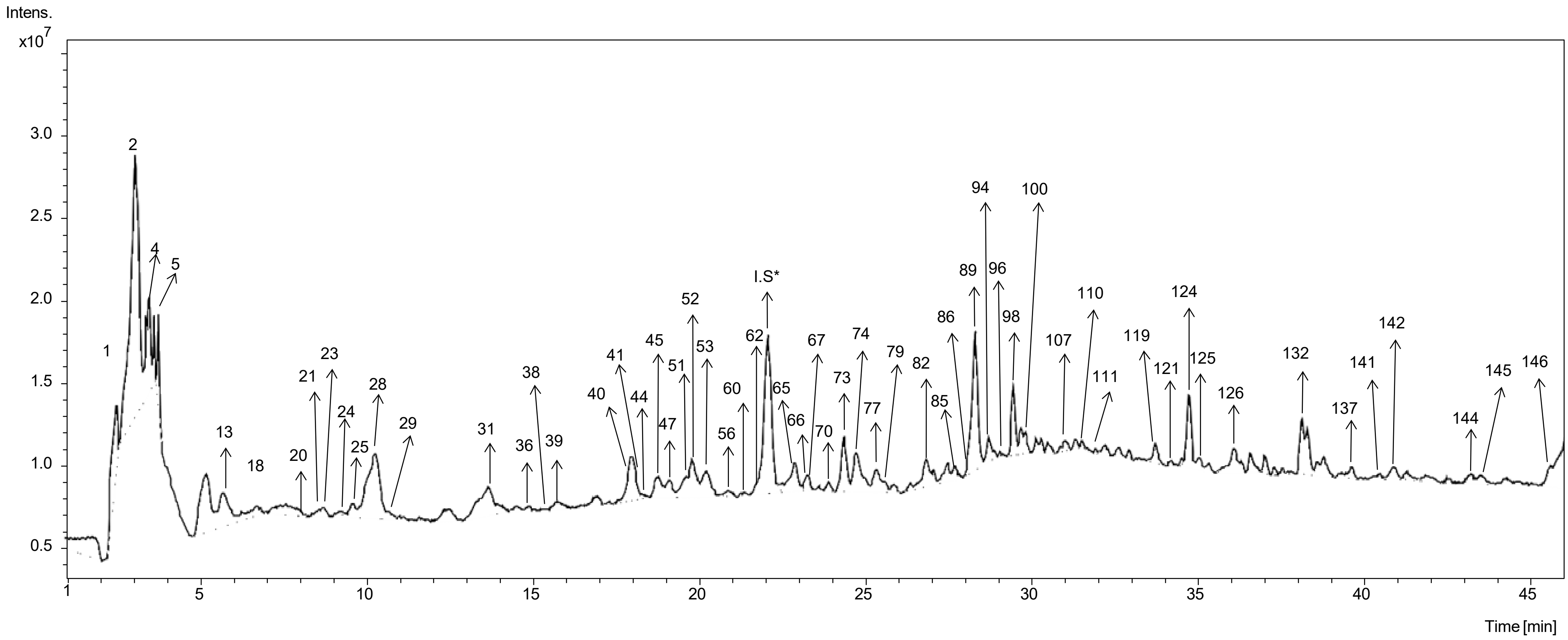

Figure S2. Base-peak chromatogram of *Achillea millefolium* L. obtained by UAE (ethanol-water, 50:50, v/v) analysed by HPLC-ESI-QTOF-MS in negative ionization mode. *IS, internal standard (ethyl gallate).
